# Role of delayed-time-point imaging during abdominal and pelvic cancer screening using FDG-PET/CT in the general population

**DOI:** 10.1097/MD.0000000000008832

**Published:** 2017-11-17

**Authors:** Shotaro Naganawa, Takeharu Yoshikawa, Koichiro Yasaka, Eriko Maeda, Naoto Hayashi, Osamu Abe

**Affiliations:** a Department of Radiology, Graduate School of Medicine, The University of Tokyo; b Department of Computational Diagnostic Radiology and Preventive Medicine, The University of Tokyo Hospital, Bunkyo-ku; c Department of Radiology, The Institute of Medical Science, The University of Tokyo, Minato-ku, Tokyo, Japan.

**Keywords:** abdomen and pelvis, cancer screening, computed tomography, dual-time phase image, FDG-PET

## Abstract

Although delayed-time-point imaging is expected to improve the results of [^18^F]-fluorodeoxyglucose (FDG)-positron emission tomography/computed tomography (PET/CT), how examinees will benefit from dual-time-point imaging versus initial-time-point imaging alone, remains unclear. This study investigated the role of delayed-time-point imaging in improving the results of abdominal and pelvic cancer screening using FDG-PET/CT.

This retrospective review included 3131 screening results (average subject age: 55.5 years, range: 40–88 years). First, 2 nuclear medicine physicians tentatively evaluated whole-body initial-time-point PET/CT scans. Subsequently, delayed-time-point imaging of the abdomen and pelvis was performed approximately 150 min after FDG injection, followed by re-evaluation for necessary changes. All changed records were retrospectively reviewed and classified as either lesions that were found in initial-time-point images but were changed into negative by adding delayed scan or newly detected findings of suspected malignancy on delayed-time-point images; lesions suspected to be malignant were subjected to further pathologic review. Diagnostic performance according to sensitivity, specificity, accuracy, positive predictive value (PPV), and negative predictive value (NPV) were calculated and compared between initial-time-point and dual-time-point imaging.

Fifty-four records were changed after addition of the delayed-time-point imaging. Of the 105 suspected malignancies on initial-time-point images, 10 were changed into negative following the delayed scan. In addition, 44 lesions were newly detected as suspected malignancies on delayed-time-point images. Thirty-six lesions were proven to be pathologically malignant. Of these, 26 were detected on initial-time-point images, and 8 lesions (gastrointestinal adenocarcinoma, 6; prostate adenocarcinoma, 2) were observed on delayed-time-point images. The sensitivity of dual-time-point imaging (58.6% [34/58]) was significantly higher than that of initial-time-point imaging only (44.8% [26/58]) (*P* = .005); however, specificity and accuracy of dual-time-point imaging (96.6% [2968/3073] and 95.9% [3002/3131], respectively) were significantly lower than those of initial-time-point imaging only (97.4% [2994/3073] and 96.5% [3020/3131], respectively) (*P* < .0001 and *P* = .013, respectively). There were no significant differences in PPV (initial-time-point imaging: 24.8% [26/105], dual-time-point imaging: 24.5% [34/139]) and NPV (98.9% [2994/3026] and 99.2% [2968/3073], respectively).

The inclusion of delayed PET/CT in screening examinations facilitated the detection of pathologically malignant lesions, particularly in the gastrointestinal tract, while also detecting benign and false-negative lesions.

## 1 Introduction

Positron emission tomography/computed tomography (PET/CT) with [^18^F]-fluorodeoxyglucose (FDG) is widely used in assessing cancer biology and in staging of cancers and treatment monitoring of cancer patients.^[1]^ As FDG-PET/CT is can detect unexpected cancers in many organs in a single examination,^[2,3]^ it is also used in cancer screening. Past literature shows a detection rate of 1.0% to 3.3% in a population screened using whole-body PET/CT,^[3–5]^ but the sensitivity and specificity of this technique are limited. Nishizawa et al reported that the sensitivity and specificity were 81.8% and 82.0%, respectively.^[3–5]^ Delayed-time-point imaging techniques have been introduced to improve the sensitivity and specificity of PET/CT screening. On delayed-time-point images, the background radioactivity decreases over time as a result of the continued FDG clearance from normal tissues^[6]^; by contrast, most malignant lesions exhibit stable or increased FDG uptake over time, thus increasing the lesion-to-background ratios and facilitating the identification of significant lesions.^[7]^

Physiologic levels of FDG uptake are often observed in the gastrointestinal tract because of smooth muscle activity, metabolically active mucosa, and colonic microbial uptake.^[8]^ However, some areas of physiologic FDG uptake decreased or changed in shape and/or location, whereas all areas of pathological FDG uptake remained visually unchanged.^[9]^ As we were able to exploit this difference to reduce the incidence of false-positive findings, dual-time-point imaging, which includes “initial” and “delayed” time-point imaging, would be expected to improve the sensitivity and accuracy of FDG-PET/CT screening. However, consensus is lacking regarding the usage of this technique, and how examinees would benefit from dual-time-point imaging versus initial-time-point imaging alone remains unclear.^[7]^ Therefore, this study aimed to investigate whether the addition of delayed time-point imaging to FDG-PET/CT cancer screening improved the results relative to those obtained using initial-time-point imaging alone.

## 2 Materials and methods

### 2.1 Subject population

This study was approved by our institutional review board. All data were retrieved from a database of our institutional comprehensive health screening program, and all examinees had been recruited from the general public. Before participating in the program, all examinees provided written informed consent for the storage of their clinical, laboratory, and imaging data in a database and the use of these data for research purposes. All procedures involving human participants were performed in accordance with our institutional ethical standards and the 1964 Helsinki declaration and its later amendments. A retrospective review of our registry database yielded 18,125 examinees enrolled from April 2008 to March 2016, including 5663 examinees who underwent FDG-PET/CT for the first time. Examinees without follow-up data were excluded, and the remaining 3131 examinees (mean age, 55.5 ± 10.9 years; range, 40–88 years) constituted the study group.

### 2.2 FDG-PET/CT image acquisition

PET/CT scans were performed using a Discovery ST Elite scanner (General Electric Medical Systems, Milwaukee, WI). Initial PET/CT scan covered the neck, thorax, abdomen, and pelvis, while delayed PET/CT scan covered the abdomen and pelvis. All examinees fasted for at least 5 h before the imaging study, and blood glucose levels were measured immediately before FDG injection (mean blood glucose level = 99.6 ± 18.0 mg/dL). Examinees rested in a dark room for 50 min after receiving an intravenous dose of FDG (3.7 MBq/kg). CT images were then acquired using a 16-detector row scanner with the following parameters: 30 to 210 mA, depending on an anatomic location, using an automatic exposure control system; 120 kV, 0.5 s/rotation, 3.75-mm section thickness, 512 × 512 matrix, and 1.75:1 pitch factor. The initial PET emission scan was performed with the following parameters: 128 × 128 matrix; 3.3-mm interval; 2 iterations, and 14 subsets. There were no restrictions after the initial PET scan. Approximately 150 min after FDG injection, low-dose CT images for delayed-time-point imaging were performed with the following parameters: 40 mA, 120 kV, 0.5 s/rotation, 3.75-mm section thickness, 512 × 512 matrix, and 1.75:1 pitch factor; delayed PET scans were performed with following parameters: 128 × 128 matrix, 3.3-mm interval, 2 iterations, and 14 subsets. For delayed-time-point imaging, the dose-length product (DLP) was 59.4 mGy-cm, and the effective dose, estimated by multiplying DLP by the weighting factor of 0.015, was 0.89 mSv.^[10]^

### 2.3 Image interpretation

First, the initial-phase PET/CT images were evaluated and tentative reports were issued. Next, delayed-phase PET/CT imaging was performed, and the images were evaluated, after which the tentative reports were revised as needed to yield final reports. All PET/CT images were independently evaluated by 2 nuclear medicine physicians, and the final diagnosis was reached by consensus when the readers’ diagnoses differed. The initial- and delayed-phase PET/CT images were basically evaluated by the same readers. In total, 33 board-certified nuclear medicine physicians who had at least a 5-year experience of PET/CT (average 7.2 ± 2.9 years; range 5–20 years) participated in image interpretation. Readers referred to examinee's blood test results including carcinoembryonic antigen, carbohydrate antigen 19-9, prostate-specific antigen (PSA), and carbohydrate antigen 125 as needed.

The past literature showed a difference between pathological and physiological lesions; specifically, pathological lesions remained visually unchanged on delayed-time-point images, whereas physiological lesions could change shape or location.^[9]^ Thus when lesion signals detected on initial-phase PET/CT images changed in shape or extinguished on delayed-phase PET/CT images, they were considered to indicate physiological uptake and were removed from the final reports, whereas lesions detected only on delayed-phase PET/CT images were recorded as newly detected lesions in final reports. No definitive maximum standard uptake value (SUV max)-based criterion was introduced. Instead, decisions regarding whether to mark a lesion as positive were left to the readers’ discretion.

Detected lesions were classified into 1 of 4 categories: “benign” if a benign lesion was suspected; “probable benign” if the lesions appeared benign but annual follow-up was recommended; “potential malignancy” when further examination or close follow-up was recommended; and “emergency” when emergency treatment was recommended.

### 2.4 Evaluation of outcomes

Examinees with lesions classified as “potential malignancy” and “emergency” were referred to appropriate hospitals for definitive diagnosis and necessary treatment. We followed up these examinees by contacting the referred hospitals or at the next annual screening. Outcomes were classified into 1 of 4 categories: “malignant” when lesions were demonstrated to be pathologically malignant; “benign” when further examination revealed benign lesions or no interval change was observed during the next annual screening; “false positive” when the lesions were not observed in subsequent examinations or the next annual screening; “false negative” when malignant lesions were found within a year after the examination; and “true negative” when no malignant lesions were found within a year after the examination.

### 2.5 Statistical analysis

All tentative and final reports were reviewed and revision histories were extracted. Histories of change were closely reviewed and classified as either lesions detected on initial PET/CT images but changed into negative by adding delayed scan, or newly detected lesions on delayed PET/CT images. The sensitivity, specificity, accuracy, positive predictive value (PPV), and negative predictive value (NPV) for malignancy were calculated using pathological results as a reference standard. The sensitivities, specificities, and accuracies between the results of initial PET/CT only and dual-phase PET/CT were compared using McNemar test, and the PPVs and NPVs were compared using Fisher exact test. Malignant lesions known before the examination were excluded from the statistical analysis.

Data are presented as averages ± standard deviations. A *P* value of <.05 was considered to indicate a statistically significant difference. All statistical analyses were performed using the JMP v.11.2.0 software (SAS Inc., Cary, NC).

## 3 Results

**Figure 1:**
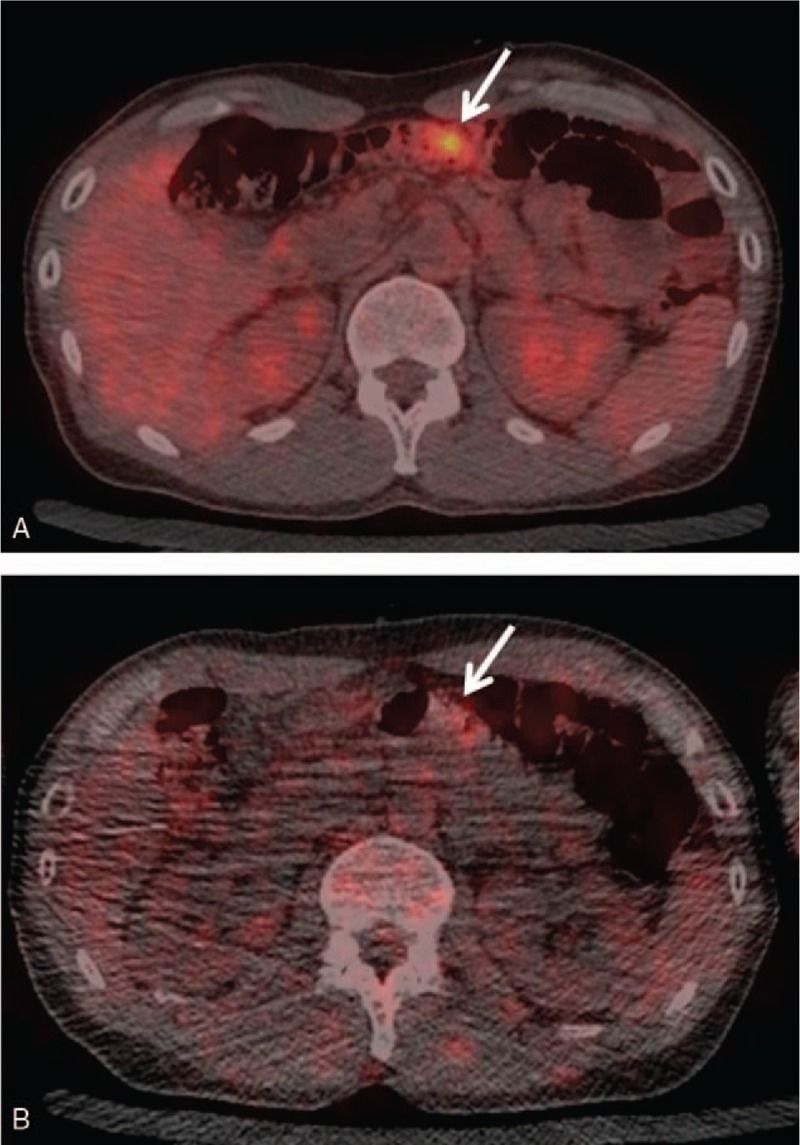
Axial [^18^F]-fluorodeoxyglucose (FDG)-positron emission tomography/computed tomography images of a 42-year-old man. Nodular uptake (maximum standard uptake value: 17.5) in the transverse colon was observed on an initial-time-point image (arrow) (A), but disappeared on a delayed-time-point image (arrow) (B). The lesion was considered to be physiological FDG uptake.

**Figure 2:**
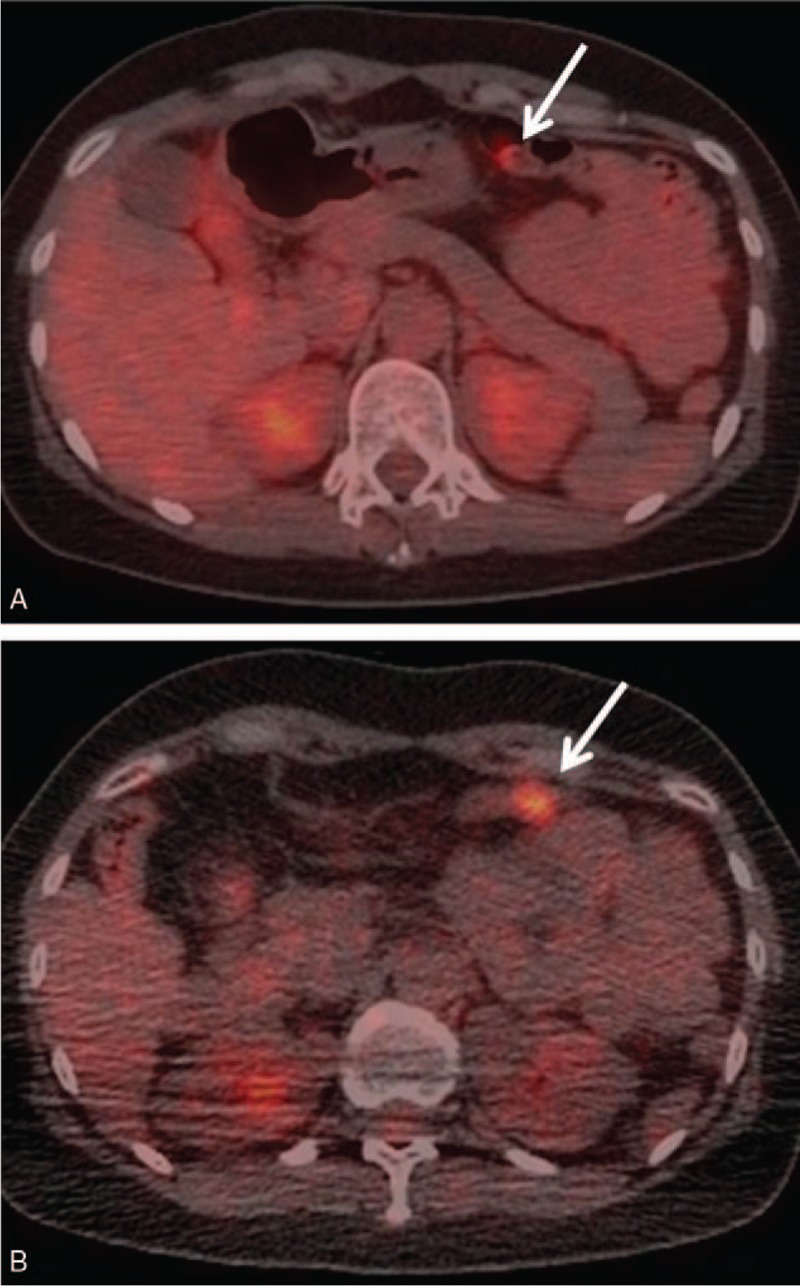
Axial [^18^F]-fluorodeoxyglucose (FDG)-positron emission tomography/computed tomography images of a 48-year-old woman. On the initial-time-point image, FDG uptake (maximum standard uptake value [SUV max]: 13.6) in the transverse colon was ambiguous and difficult to distinguish from the background or physiological uptake (arrow) (A). Nodular uptake (SUV max: 8.4) was definitive on a delayed-time-point image (arrow) (B). The lesion was pathologically proven to be adenocarcinoma.

**Figure 3:**
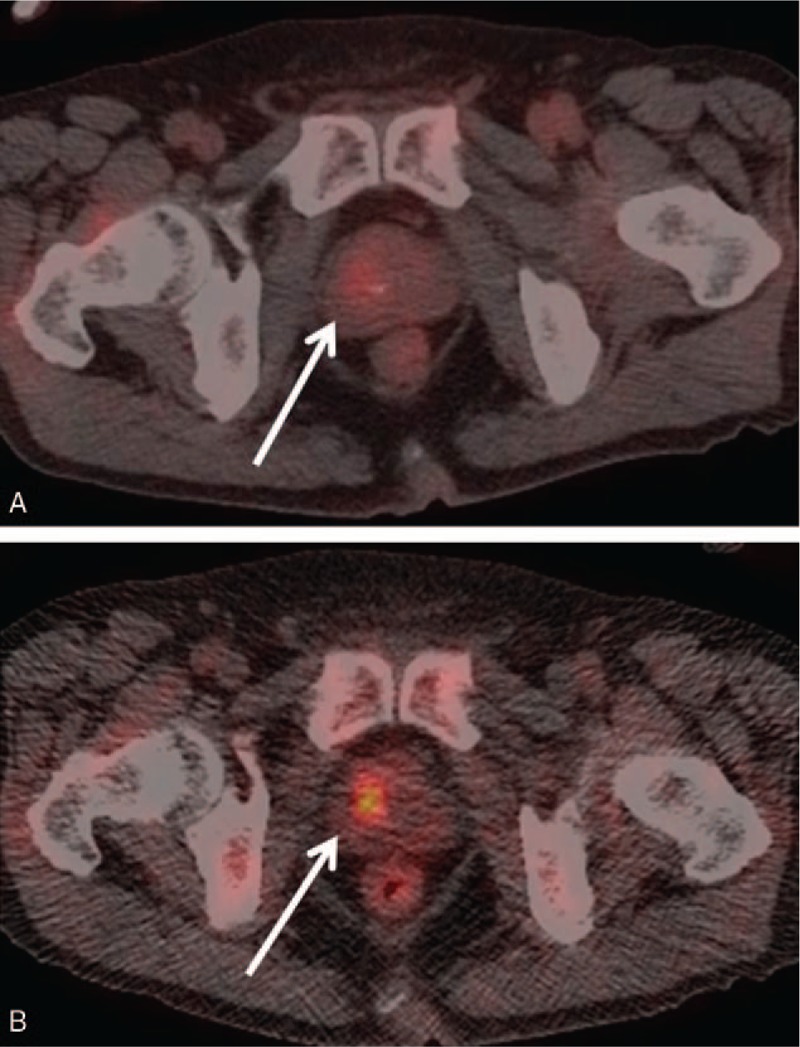
Axial [^18^F]-fluorodeoxyglucose (FDG)-positron emission tomography/computed tomography images of a 72-year-old man. On the initial-time-point image, FDG uptake (maximum standard uptake value [SUV max]: 10.4) in the prostate was ambiguous and difficult to distinguish from the background (arrow) (A). Definitive nodular uptake (SUV max: 9.2) in the right lobe of the prostate was observed on the delayed-time-point image (arrow) (B). The lesion was pathologically proven to be adenocarcinoma.

The study cohort consisted of 2113 men (average subject age: 56.6 ± 10.6 years, range: 40–88 years) and 1018 women (average subject age: 55.5 ± 10.4 years, range: 40–85 years). A total of 149 malignancy-suspected lesions were detected on initial- and delayed-time-point imaging in 148 examinees including 9 known malignant lesions (liver metastases from breast cancer, bone metastases from rectal cancer, bone metastases from breast cancer, renal metastasis from breast cancer, renal metastasis from hypopharyngeal carcinoma, lymph node metastasis of gastric cancer, peritoneal dissemination of gastric cancer, prostate cancer, and status postretroperitoneal). Table 1 lists the lesions detected using initial-time-point imaging and their outcomes. These lesions included 10 lesions that were found in initial-time-point images but were changed into negative by adding delayed scan (stomach: 3, ileum: 2, colon: 3, rectum: 1, prostate: 1) (Fig. 1). All of these 10 lesions were confirmed to be nonmalignant during the next annual screening. Newly detected lesions on delayed-time-point images are listed in Table 2. Thirty-six of the lesions found in dual-time-point imaging were proven to be pathologically malignant. Of these, 26 (gastrointestinal tract: 11, hepato-biliary-pancreas: 4, urogenital: 8, others: 3) were detected on initial-time-point images alone (Table 3), and 8 (gastric adenocarcinoma: 1, colon adenocarcinoma: 3 [Fig. 2], rectal adenocarcinoma: 2, prostate adenocarcinoma: 2 [Fig. 3]) were detected on delayed-time-point images.

**Table 1:**
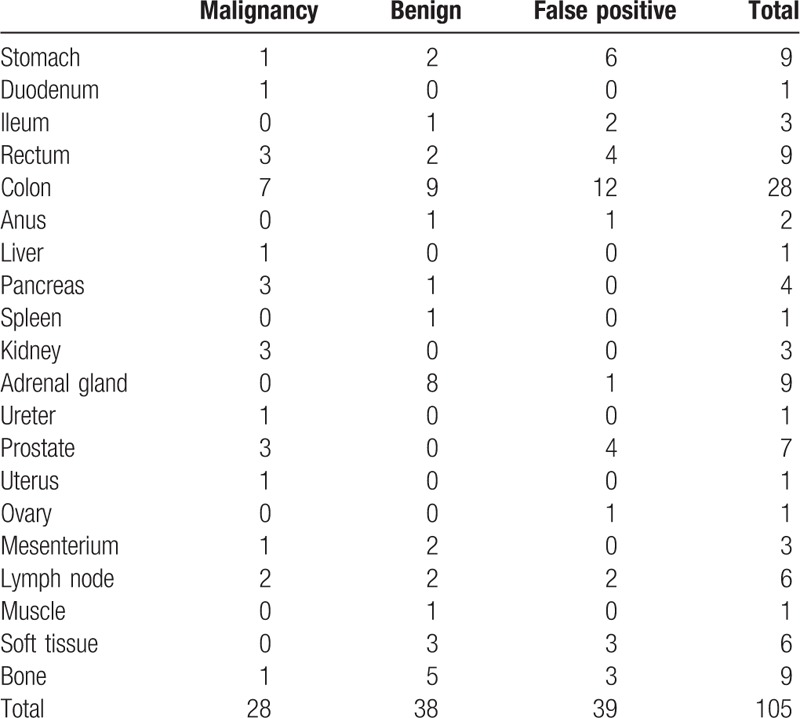
Outcomes of lesions detected using initial-time-point imaging.

**Table 2:**
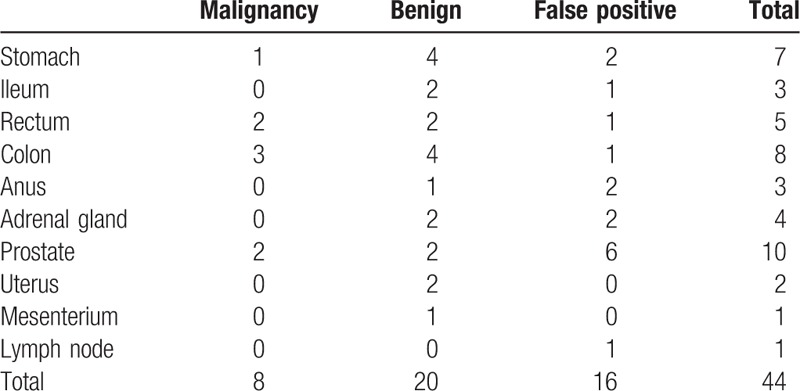
Outcomes of newly detected lesions on delayed-time-point images.

**Table 3:**
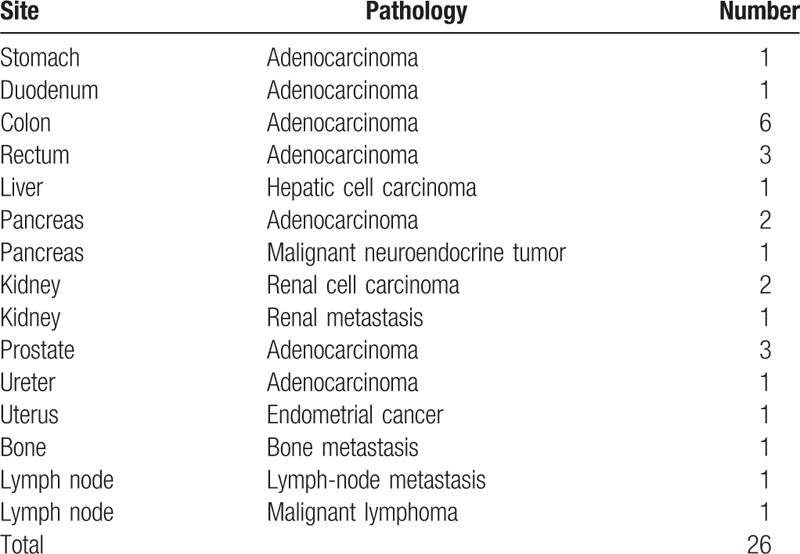
Malignant lesions detected on initial-time-point positron emission tomography/computed tomography images alone.

Within 1-year follow-up after the initial examination, 24 interval cancers were found to have developed malignant lesions (hepatocellular carcinoma: 3, renal cell carcinoma: 5, gastric adenocarcinoma: 3, colon adenocarcinoma: 6, rectal adenocarcinoma: 1, cervical cancer: 1, and prostate adenocarcinoma: 5).

The diagnostic performances are shown in Table 4. The sensitivity of dual-time-point imaging (58.6% [34/58]) was significantly higher than that of initial-time-point imaging only (44.8% [26/58]) (*P* = .005); however, specificity and accuracy of dual-time-point imaging (96.6% [2968/3073] and 95.9% [3002/3131], respectively) were significantly lower than those of initial-time-point imaging only (97.4% [2994/3073] and 96.5% [3020/3131], respectively) (*P* < .0001 and *P* = .013, respectively). There were no significant differences in PPV (initial-time-point imaging: 24.8% [26/105], dual-time-point imaging: 24.5% [34/139]) and NPV (98.9% [2994/3026] and 99.2% [2968/3073], respectively).

**Table 4:**
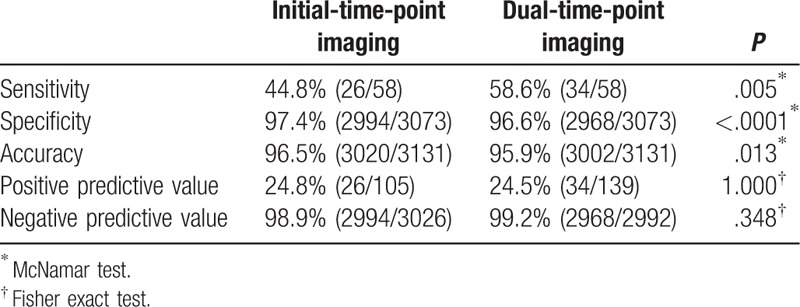
Comparison of diagnostic performance between initial-time-point and dual-time-point imaging.

## 4 Discussion

This study intended to compare the results of initial-time-point imaging and dual-time-point imaging with the aim of clarifying the value of added delayed-time-point imaging. Our results demonstrated that the addition of delayed-time-point imaging significantly increased the sensitivity of FDG-PET/CT imaging for malignant lesions by 13.8%, while the specificity and accuracy significantly decreased by <1%. The PPV and NPV of FDG-PET/CT imaging for malignant lesions were not improved by adding delayed-time-point imaging. Notably, this was the first study in which initial- and delayed-time-point imaging were evaluated sequentially in a large population.

Ten lesions detected on initial-time-point PET/CT images were changed from positive to negative by the addition of delayed-time-point images. Of these, 9 lesions were identified in the gastrointestinal tract, a phenomenon attributed to the physiologic FDG uptake as a result of smooth muscle activity, metabolically active mucosa, and colonic microbial uptake. Notably, a previous study concluded that 30% of incidental focal uptake was physiologic.^[11]^ Miyake et al^[9]^ demonstrated differences between pathological and physiological lesions, whereby pathological lesions remained visually unchanged on delayed-time-point images while physiological lesions could change shape. Certainly, by comparing initial-time-point imaging with delayed-time-point imaging, it is easy to distinguish pathological uptake from the peristaltic movement of FDG excreted into the bowel.

Among the 8 malignant lesions observed only on delayed-time-point images, 6 (majority) were found in the gastrointestinal tract and 2 were found in the prostate. Of the former, background radioactivity decreased over an 8-h washout period,^[12]^ whereas high FDG uptake was maintained in malignant lesions.^[9]^ This increasing contrast between the lesion and background enabled identification of lesions that remained hidden on the initial-time-point images.

Although 10 prostate lesions were newly detected on delayed-time-point images, only 2 were pathologically malignant. According to a previous study, PPV of PET/CT for prostate cancer was 20%,^[13]^ while another study showed that 95% of focal prostate FDG uptake was benign; however, a lesion in the peripheral zone without coincidental calcification suggested an existing malignancy.^[14]^ We assume that it is difficult to exclude all lesions exhibiting FDG uptake for screening purposes, whereas it would be reasonable to exclude lesions with calcification that are suspected as benign. In addition, PSA has a higher sensitivity for prostate cancer and should be included in cancer screening.^[13]^ In our study, readers prepared the reports independent of the serum level of PSA because PSA alone could not help detect 10% of prostate cancers.^[15]^

Eight malignant lesions were newly detected on delayed-time-point images and the sensitivity was significantly improved by 13.8%. However, at the same time, many benign and false-positive lesions were also detected as suspected malignant lesions. As a result, specificity and accuracy significantly deteriorated and there were no difference in PPV and NPV of FDG-PET/CT imaging for detection of malignant lesions. Although the specificity and accuracy of dual-time-point imaging were significantly lower than those of initial-time-point imaging, the differences were quite subtle (<1%) and likely clinically irrelevant compared with the improved sensitivity. The benign and false-negative lesions detected on delayed-time-point images were distributed to wide range of organs. As for colorectal lesions, Minamimoto et al showed that increased FDG uptake on delayed-time-point image compared with initial-time-point image tended to indicate a high probability of cancer (PPV = 40.3%), whereas stable and decreased uptakes had smaller PPV (18.9% and 20.5%, respectively)^[16]^; this tendency may contribute to the improvement of PPV, but it is difficult to exclude lesions exhibiting FDG uptake for screening purposes.

Additional radiation exposure (low-dose CT) and extra time were needed to apply delayed-time-point imaging. Although the estimated effective dose was only 0.89 mSv, an amount not expected to cause acute harm to human health, the potential risk of carcinogenesis is unknown. According to the linear no-threshold hypothesis (LNTH), carcinogenesis is directly proportional to the radiation dose, even at doses <1 mSv. However, the American Nuclear Society stated that there was insufficient evidence to support the LNTH with respect to the health effects of low-level radiation.^[17]^ In addition, the further radiation exposure incurred during this procedure is lower than the estimated worldwide average annual exposure from natural sources (2.4 mSv).^[18]^ We cannot clearly state whether this extra low-dose radiation exposure could be justified by the increased malignancy detection rate observed in this study, but we believe that this record will facilitate further consideration.

In this study, we performed delayed-time-point imaging approximately 150 min after FDG injection. One previous report recommended an interval of at least 2 h and ideally 3 h after FDG injection,^[7]^ whereas another report demonstrated a higher diagnostic value when imaging was performed 110 min after FDG injection, compared with 233 min after FDG injection.^[19]^ We assumed that 150 min was reasonable with regard to both the situation and previous literature, while considering the examinees’ comfort.

This study has several limitations that should be addressed. First, we did not set a definitive SUV-related threshold or criterion. However, it was difficult to determine the definite criteria because decisions regarding whether to record lesions should be based on several factors, including SUV max, physiological situation, and shape and site of FDG uptake. Second, many readers with varying levels of experience participated in this screening program; however, one might argue that this factor enhanced the generalizability of our findings. Third, although the initial-time-point images were evaluated before the delayed scans were performed, there was a possibility that the initial reading was not performed carefully because the readers knew that the images would be interpreted together with the delayed images to prepare the final report. Fourth, this study was retrospectively designed to include results from referred hospitals or subsequent annual screening. Accordingly, the outcomes of some examinees were not followed up. Fifth, as this health screening program was originally designed for the general public except critically ill patients, the study group included some examinees that were previously diagnosed with cancer. Nine examinees actually had abnormal FDG uptake related to the previously diagnosed cancer, and some other examinees were also expected to have a history of cancer. Moreover, all examinees voluntarily participated in this health screening program for the purpose of detecting latent diseases and preventing an asymptomatic disease from progressing to symptomatic disease at their own expense. Therefore, this study group might have had relatively higher health consciousness than the general population.

In summary, the sensitivity for malignant lesions increased by 13.8% after adding delayed-time-point imaging to screening procedures. However, the specificity and accuracy slightly decreased, and the PPV and NPV were not improved. Most malignant lesions identified using delayed-time-point imaging were found in the gastrointestinal tract.

## Acknowledgment

The authors thank to Dr. Francis Ha at Monash University, Australia for confirming that our manuscript is written in correct scientific English.
